# Failure rates associated with guided versus non-guided dental implant placement: a systematic review and meta-analysis

**DOI:** 10.1038/s41405-021-00086-1

**Published:** 2021-08-18

**Authors:** Nancy Abdelhay, Soni Prasad, Monica Prasad Gibson

**Affiliations:** 1 Faculty of Medicine and Dentistry, Department of Dentistry, University of Alberta, Edmonton, AB Canada; 2 Faculty of Dentistry, Alexandria University, Alexandria, Egypt; 3 Graduate Prosthodontics, Marquette University School of Dentistry, Milwaukee, WI USA; 4 Faculty of Medicine and Dentistry, Division of Periodontology, University of Alberta, Edmonton, AB Canada

**Keywords:** Dental implants, Fixed prosthodontics

## Abstract

**Objective:**

The purpose of the systematic review and meta-analysis was to evaluate implant failure rates and their association with guided and free-hand implant placement techniques.

**Materials and methods:**

A literature search was conducted across PubMed, Medline via Ovid, Cochrane database, and Google Scholar. The search was completed in September 2020. Series of meta-analyses were conducted to compare implant failure rates with guided and free-hand techniques.

**Results:**

A total of 3387 articles were identified from the electronic search. After applying the inclusion criteria, eight articles were selected for qualitative assessment and four for quantitative synthesis (meta-analysis). The included studies had a risk ratio of 0.29 (95% CI: 0.15, 0.58), *P* < 0.001 for the use of guided implant placement. Implant failure rates were affected by the different placement techniques indicated by the test for overall effect (*Z* = 3.53, *P* = 0.0004). The incidence of implant failure in guided surgery versus free-hand surgery was found to be 2.25% and 6.42%, respectively.

**Conclusion:**

Both guided and free-hand implant placement techniques resulted in a high implant survival rate. However, implant failure rates were almost three times higher in the free-hand implant placement category. A guided implant placement approach is recommended for a successful outcome.

## Introduction

Prosthodontic rehabilitation with dental implants requires accurate implant placement for predictable functional, and aesthetic outcomes.^1,2^ Implant dentistry has developed numerous advancements in technology, materials, techniques, and concepts to achieve the desired beneficial clinical results.^3^

Implant placement is a prosthetically driven procedure and requires thorough restorative and surgical treatment planning.^4^ The patient’s anatomy, medical condition, practitioner experience, and surgical approach are factors that ultimately influence the outcome.^5,6^ There are several surgical techniques utilized during implant placement.^7^ A surgeon can place an implant either free-hand, with a pilot drill guide, or with a fully guided system.^8,9^ A fully guided template is fabricated with the help of a cone-beam computed tomography (CBCT) and related implant planning software.^10,11^ This process involves a “crown-down” approach that visualizes an ideal prosthetic position to orient the virtual implant placement.^12,13^ A surgical guide is then fabricated to replicate this planned position.^14^ The CBCT also provides an in-depth understanding of the underlying bone morphology, anatomy, and location of vital structures.^2,15^

Recently, a dynamic navigation system has been introduced to aid in implant placement. This technology uses intraoperative optical tracking of the hand-piece position with installed cameras to guide the surgeon in “real-time,” thus providing visual feedback on a screen.^16^ However, this review will focus on the comparison between free-hand and fully guided implant placements only.

The fully guided implant placement that utilizes the computer-generated guide is more accurate than traditional surgical guides and free-hand placements.^17^ Several excellent studies and reviews have outlined the accuracy of computer-generated guides with respect to anatomical landmarks and range of error.^18–20^ However, not many reviews have investigated implant failures.

Once an implant is placed, the outcome of its placement is dependent on distinct clinical and radiographic parameters that determine the implant’s success or survival.^21^ A “successful” implant is defined as an implant remaining in situ and free of all biological and technical complications over the entire observation period.^22^ According to Smith and Zarb, implant success criteria include the absence of mobility, peri-implant radiolucency, pain, and infection. In addition, annual bone loss should be <0.2 mm after the first year of service along with the satisfactory appearance of implant prostheses. They proposed a minimum success rate of 85% at the end of 5 years and 80% at the end of 10 years.^23^ On the other hand, implant survival refers to the implant remaining in situ at the follow-up examination.^22,24^

The goal of surgical implant placement is to have a high success rate. However, a surviving implant that does not meet all the success criteria is sometimes an acceptable (but not ideal) outcome. Early implant failure resulting primarily from inaccurate planning or surgical complications causes angst among patients and surgeons alike.^25^ Also, previously failed implant sites are at a higher risk for future implant placement.^26^ Therefore, attempts should be made to avoid early implant failures. Implant success and survival rely on the osseointegration between the implant surface and surrounding bone.^27^ Lack or loss of osseointegration results in implant failures.^28,29^ Early implant failures occur before prosthetic loading and are seen in 1–2% of patients within the first few weeks.^30^ Late failures usually occur after prosthetic loading and are seen in about 5–10% of patients.^31^ Peri-implantitis, factors affecting the microbial environment, and prosthetic rehabilitation are common causes for late implant failure.^32^

While several excellent reviews have compared the success and survival rates between free-hand versus fully guided implant placement, very few have evaluated the failures.^28,29,32–34^ Researchers have correlated possible early and late implant failure risk factors with age, sex, smoking,^35^ type of edentulism, bone quality and volume,^36^ implant location, diameter, length,^37^ immune factors, and various systemic diseases.^38^ Although computer-guided implant placement is predictable, its performance with respect to failure rates must be critically evaluated and compared with free-hand implant placement. This systematic review aimed to evaluate the association between implant failure rates and surgical placement using fully guided and free-hand techniques.

## Materials and methods

### Study selection

A literature search was performed in September 2020 across four electronic databases: PubMed and Medline via Ovid (1990 to September 2020), Cochrane database (Wiley, September 2020), and Google Scholar. No time or language restrictions were applied to attain the maximum number of results regarding implant dentistry. Manual record search across dental journals and other relevant databases generated literature more specific to the review focus and undiscovered from the above primary databases. Several combinations of keywords like dental implants, guided, non-guided, free-hand, three-dimension, peri-implantitis, and risk factors, were used during the search process to generate records pertaining to implant failure and use of surgical guides. Primary screening involved examining the title and abstract of generated records. Full-text studies that seemed to meet the criteria were included and further assessed. Two reviewers independently carried out the secondary screening of the remaining records involving full-text assessment of study methods, results, and discussions. The final selection of studies was made by a discussion between authors under PICO-based inclusion/exclusion criteria.

### Inclusion, exclusion criteria and study design (PICO)

The Population of interest included all patients in need of implant placements and patients referred/scheduled for implant surgery. General inclusion criteria were healthy adults >18 years of age who were nonsmokers or light smokers (<5 cigarettes a day). Interventions were oral implant placements. The Comparison involved the usage of guided versus free-hand implant placements. Experimental studies directly pertaining to the Outcome in question were examined for data extraction. Included study types were randomized controlled trials (RCTs), clinical controlled trials (CCTs), and prospective/retrospective clinical studies. For the meta-analysis, included study designs were RCTs and CCTs. Outcomes of interest were (1) the incidence of early implant failure using either technique and (2) associated risk factors. Subsequently, patients were excluded if they were immunocompromised, <18 years of age, pregnant, or had systemic disease. Studies without treatment interventions such as reviews, case reports, and commentaries were excluded. Finally, studies were excluded if they did not report on implant failure outcomes or had different populations of interest.

### Assessment based on Quality Appraisal of Reliability Studies (QAREL) Checklist

The risk of bias assessment for included RCTs and cohort studies was performed following the guidelines by Cochrane systematic review handbook.^39,40^ JBI Critical Appraisal Checklist for Quasi-Experimental Studies for non-randomized and randomized experimental studies was used.^41^

The QAREL Checklist 28 was used on the included studies to identify their reliability according to 11 items. The QAREL Checklist provided a quality assessment for the spectrum of participants and examiners, examiner blinding, order effects of examination, suitability of the time interval among repeated measurements, appropriate test application and interpretation, and appropriate statistical analysis. The checklist also helped assess the variability in performance and reporting of physical examination procedures. Each criterion was framed as a question and had response options of definitely yes (low risk of bias), probably yes, probably no, and definitely no (high risk of bias). The included studies’ quality was classified based on QAREL scores: a score of 67% or more indicated high quality, 50–66% moderate quality, and <50% low quality.

### Data extraction

The data extraction process was executed in duplicate and independently by two authors. It was then double-checked between two authors to validate the gathered information. Any disagreements were resolved by discussion or consultation with the third author.

### Data synthesis and statistical analysis

The meta-analysis was performed using RevMan 5.3, constructing a forest plot with *I*^2^ statistics to analyze and present variability due to heterogeneity among the gathered studies. Relative weights of included studies were expressed in percentages, and the risk ratio (RR) with 95% confidence interval (CI) was computed per study or subgroup.

## Results

### Literature search

**Fig. 1:**
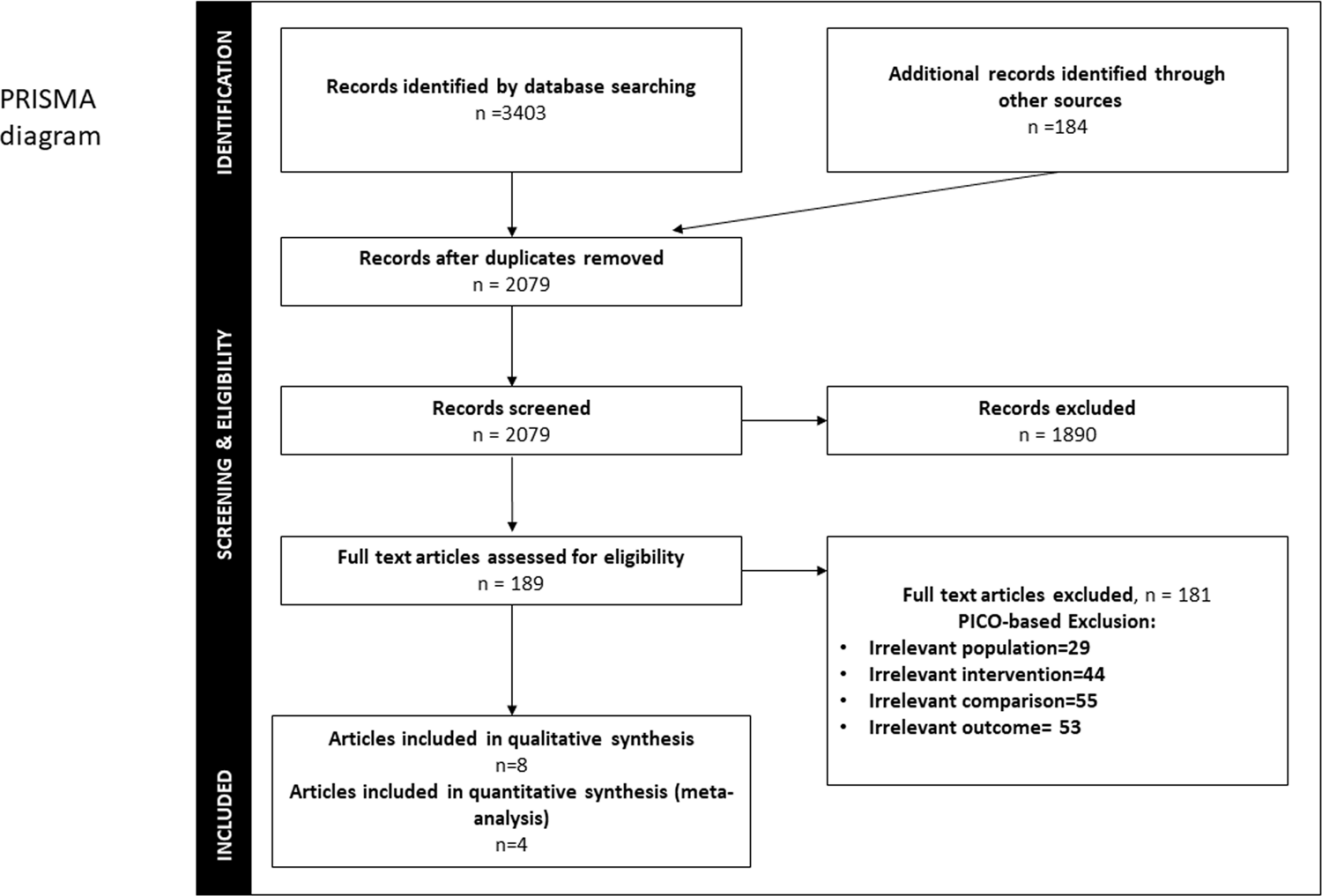
PRISMA flow diagram of literature search strategy: including identification, screening, eligibility examination, and final inclusion. The number of records identified during the initial search represents the sum of all papers collected through each electronic database.

A total of 3403 potentially relevant titles and abstracts were found by the electronic search and additional evaluation of reference lists. During the first screening, 2079 publications were excluded after duplicates were removed. Another 1890 records were excluded based on the title and keywords, and abstract evaluation. In addition, 189 full-text articles were thoroughly evaluated. A total of 181 papers were excluded because they did not fulfill the systematic review’s inclusion criteria (Fig. 1). Eight articles were included in the qualitative assessment and four in quantitative synthesis (meta-analysis).

### Description of studies

A detailed summary of the included studies is presented in Table 1. Data extraction identified three RCTs^4,42,43^ and five cohort studies.^6,44–47^ All studies stated the number of implants placed using guided versus free-hand techniques and the investigation outcomes. Five studies^4,43,45–47^ identified implant failure as it is related to guided or free-hand implant placement. One study investigated the malpositioning of implants placed using either mucosa- or bone-supported guides and free-hand placement.^44^ One RCT study investigated free-hand implant placement with preoperative CBCT and postoperative periapical radiograph.^6^ Another study compared free-hand and guided implant placement by prosthodontists and maxillofacial dentists.^42^ Five studies investigated implant failure rates using any of the techniques. Two studies did not specify the number of failed implants.^15,24^

**Table 1:** Summary of included studies.

Author	Year	Type of study	Number of patients/implants	Type of placement				Findings/observational outcomes
				No. of guided implant	No. of failure	No. of non-guided	No. of failure	
Arisan et al.	2013	Cohort	54/353	169	4	184	4	Groups; SIM group (mucosa-supported guide), AYT (bone-supported group), freehand method (control). Implants emerging from the interproximal area in the SIM group (5%), AYT (16%), and control groups (19%), with (*χ*^2^ = 11.77, *P* = 0.002). Implants placed via computer-aided methods showed a higher rate of success (98% and 93% for SIM and AYT groups, respectively) in maintaining a sufficient inter-implant distance compared with implants placed via the freehand method (control group, 77%; *χ*^2^ = 12.36, *P* = 0.002). In the SIM group, all implants were parallel (within 20°) within the same segment, 2% of implants in the AYT group and 16% of implants placed in the control group were not parallel to other implants positioned in the same segment, with (*χ*^2^ = 23.34, *P* < 0.0001). Implants placed by computer-aided methods, the offset position (too lingual or buccal from the top of the alveolar crest, 11% and 13% for SIM and AYT groups, respectively) compared with the control group (7%), with (*χ*^2^ = 6.243, *P* = 0.044). Screw access holes in 36% and 48% of the implants in AYT and control groups, respectively, were improperly located, with (*χ*^2^ = 8.301, *P* = 0.015). 55%, 41%, and 16% of the implants were found to be mispositioned in control, AYT, and SIM groups, respectively, with (*χ*^2^ = 37.49, *P* < 0.001).
Behneke et al.	2012	Cohort	52/132	24	0	86	0	Groups; fully guided placement, freehand placement, freehand final drilling. Mean (max) deviation at the implant shoulder for each of freehand final drilling, freehand implant insertion, and full-guided implant insertion groups were 0.52 (0.97), 0.30 (0.78), and 0.21 (0.60) mm, respectively. At the implant apex, they were 0.81 (1.38), 0.47 (1.30), and 0.28 (0.77) mm, respectively. Significant differences seen at all aspects of measurement (implant shoulder level, apex level, and angle), yielding generally higher deviations for the freehand final drilling group. With the exception of the reduced residual dentition, at the implant tip, the transfer accuracy increased with the number of sleeve-guided site preparation steps for all kinds of templates on the freehand methods.
Choi et al.	2017	Retros-pective cohort	251/450	NA	NA	NA	NA	Groups; freehand placement with a preoperative CBCT and postoperative periapical x-ray. Average discrepancy in position and angulation between the ideal and achieved implant was calculated to quantify the accuracy of freehand implant placement. Average mesiodistal angulation discrepancy between ideal and achieved surgical placement was 5.43°, 4.57° SD. Average mesiodistal position discrepancy between ideal and achieved surgical placement was 1.13, 1.48 mm SD. Discrepancies are within the range of those found by previous studies for freehand and guided surgery.
Danza et al.	2009	Cohort	93/300	66	NA	NA	9	Retrospective analysis by comparing a series of implants inserted with and without computer planning and custom model coordination. One hundred and seventy-six (58.6%) implants were placed in post extractive sockets; 72 (249%) were inserted in totally edentulous jaw; the antagonist was a natural tooth in 140 patients and a prosthetic device in the remaining 160 patients. Nine implants were lost, all were free hand inserted. The overall SVR was 97%. Statistically significant difference for immediately loaded implants, but multivariate analysis did not confirm the result, so no studied variable affected SVR. Kaplan–Meier method demonstrates that several variables are potentially associated to the crestal bone resorption. Implants inserted in the healed bone and in the frontal region have a better clinical outcome (i.e., lower bone resorption).
Nickenig et al.	2010	RCT	10/66	23	NA	23	NA	Groups; guided surgery, freehand (maxillofacial surgeon), freehand (prosthodontist). The accuracy of axis was significantly more precise with the 3-D surgical guide (4.2 (range, 0.0–10.0)) compared to the freehand method performed by the maxillofacial surgeon (9.8 (range, 3.7–17; *p* ¼ 0.000)) or by the prosthodontist (10.9(range, 2.0-20; *p* ¼ 0.000)). The 3-D surgical guide template produced significantly smaller variation between the planned and actual implant positions at the implant shoulder. With the freehand implantation by maxillofacial surgeon, mean deviation was 2.4 mm (0.0–7.0; *p* ¼ 0.001) in the posterior/anterior direction and 3.5 mm (4.0–7.0; *p* ¼ 0.000) in the medial/lateral direction. The comparison between the guided surgery and the freehand surgery performed by prosthodontist demonstrate a significant difference in the base-distance data (*p* ¼ 0.018 for the posterior/anterior direction; *p* ¼ 0.004 for the medial/lateral direction). The differences in base distances between freehand implantation performed by prosthodontist and maxillofacial surgeon were not statistically significant. The 3-D surgical guide template produced significantly smaller variation between the planned and the actual implant position at the apex of the implant. With the freehand implantation by the maxillofacial surgeon, these values were 2.0 mm (0.0–6.0; *p* ¼ 0.004) in the posterior/anterior direction and 2.5 mm (0.0–7.7; *p* ¼ 0.002) in the medial/lateral direction. The comparison between the guided surgery and the freehand surgery performed by the prosthodontist also demonstrated a significant difference in the base-distance data (*p* ¼ 0.003 for the posterior/anterior direction and *p* ¼ 0.002 for the medial–lateral direction). The differences in tip distances between freehand implantation performed by the prosthodontist and the maxillofacial surgeon were not statistically significant.
Pozzi et al.	2014	RCT	51/102	25	0	26	1	Groups; computer-guided implant placement aided with templates (computer-guided group) versus conventional implant placement without templates (conventional group). One provisional prosthesis failed, in the conventional group (*P* = 1.0). Four patients of the conventionally loaded groups experienced one complication each, versus five patients (six complications) in the computer-guided group (*P* = 0.726). There were no statistically significant differences between the two groups for any of the tested outcomes with the exception of more postoperative surgical pain (*P* = 0.002) and swelling (*P* = 0.024) at conventionally treated patients.
Ravida et al.	2018	Cohort	45/260	149	5	11	22	Groups; test group (computer-guided placement), control group (traditional placement). No significant difference was found between both groups in terms of biologic and technical complications, lower incidence of implant loss was observed in the test group (*P* < 0.001). A statistically significant difference in favor of the non-guided implant placement group was found for the initial cost (*P* < 0.05) but not for the prosthetic complications and total cost (*P* > 0.05).
Tallarico et al.	2018	RCT	20/62	32	0	30	1	Groups; computer-guided group or conventional freehand group. No prostheses failed during the entire follow-up. Two implants failed in the conventional group (6.6%) vs. none in the computer-guided group (*P* = 0.158). Differences between groups for implant failures and complications were not statistically significant. Five years after loading, the mean marginal bone loss was 0.87 mm ± 0.40 (95% CI: 0.54–1.06 mm) in the computer-guided group and 1.29 mm ± 0.31 (95% CI: 1.09–1.51 mm) in the freehand group. The difference was statistically significant (difference 0.42 mm ± 0.54; 95% CI: 0.05–0.75; *P* = 0.024). Patient self-reported post-surgical pain (*P* = 0.037) and swelling (*P* = 0.007) were found to be statistically significant higher in patients in the freehand group. Number of sessions from patient’s recruitment to delivery of the definitive prosthesis, number of days from the initial CBCT scan to implant placement, consumption of painkillers, averaged surgical, prosthetic, and complication times, were not statistically significant different between the groups.

### Assessment based on QAREL Checklist

The included studies’ methodological quality and reliability were assessed using the QAREL Checklist (Tables 2 and 3). The total ratings of the methodological quality of reliability or RCTs studies ranged between 50 and 100%. Among the three studies included, two studies were of high quality (Q 100%), and one study was of moderate quality (Q 50%). The risk of bias within the article is presented in Table 2. The total ratings of the methodological quality of reliability for Cohort studies ranged between 36 and 90%. Among the five studies included, three studies were of low quality (Q 36 and 54%); one was of moderate quality (Q 63%), and one was of high quality (Q 90%). The risk of bias within the article is presented in Table 3.

**Table 2:** Individual risk of bias assessment for controlled randomized studies: review authors’ judgments about each risk of bias factor among the selected studies.

Quality analysis of randomized clinical studies	Nickeing 2010	Pozzi 2014	Tallarico 2018
Is it clear in the study what is the “cause” and what’s the “effect” (i.e., there is no confusion about which variable comes first)?	y	y	y
Were the participants included in any comparisons similar?	u	y	y
Were the participants included in any comparisons receiving similar treatment/care, other than the exposure or intervention of interest?	n	y	y
Was there a control group?	y	y	y
Were there multiple measurements of the outcome both pre and post the intervention/exposure?	y	y	y
Was follow up complete and if not, were differences between groups in terms of their follow up adequately described and analyzed?	u	y	y
Were the outcomes of participants included in any comparisons measured in the same way?	u	y	y
Were outcomes measured in a reliable way?	y	y	y
Was appropriate statistical analysis used?	y	y	y
Overall appraisal:	50%	100%	100%

Seven domains were analyzed for each record, and final judgments were made by discussion between authors.

*y* yes, *n* no, *u* unclear.

**Table 3:** Individual risk of bias assessment for cohort studies: review authors’ judgements about each risk of bias factor among the selected studies.

Quality analysis of cohort studies	Danza 2009	Behneke 2012	Arisan 2013	Choi 2017	Ravida 2018
Were the two groups similar and recruited from the same population?	u	y	y	u	y
Were the exposures measured similarly to assign people to both exposed and unexposed groups?	y	y	y	u	y
Was the exposure measured in a valid and reliable way?	y	y	y	y	y
Were confounding factors identified?	n	n	n	y	y
Were strategies to deal with confounding factors stated?	y	n	n	n	y
Were the groups/participants free of the outcome at the start of the study (or at the moment of exposure)?	y	u	y	y	y
Were the outcomes measured in a valid and reliable way?	y	n	y	n	y
Was the follow up time reported and sufficient to be long enough for outcomes to occur?	y	n	n	n	y
Was the follow up complete, and if not, were the reasons to loss to follow up described and explored?	u	n	n	n	y
Were strategies to address incomplete follow up utilized?	u	n	n	n	n
Was appropriate statistical analysis used?	y	y	y	y	y
Total %	63%	36%	54%	36%	90%

Seven domains were analyzed for each record, and final judgments were made by discussion between authors.

*y* yes, *n* no, *u* unclear.

**Fig. 2:**
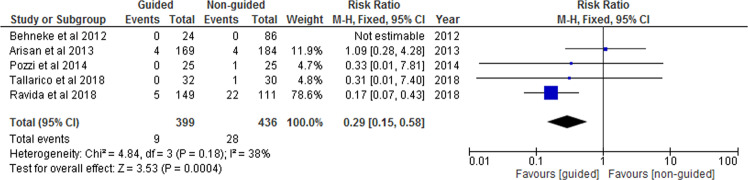
Forest Plot. Forest plot comparing the “guided” group versus “non-guided” group for the event of implant failure.

The forest plot analysis of included RCTs and cohorts is exhibited in Fig. 2. Three studies had insufficient data regarding the number of failures; hence the meta-analysis incorporated only five studies. The included studies had a RR of 0.29 (95% CI 0.15, 0.58), *P* < 0.001 for the use of guided implant placement. The forest plot exhibited medium heterogeneity (*I*^2^ = 38%). Implant failure rates were affected by the different placement techniques as indicated by the test for overall effect (*Z* = 3.53, *P* = 0.0004). According to the results, the incidence of implant failure in guided surgery versus non-guided is 2.25% and 6.42%, respectively. There was no statistically significant heterogeneity observed (*I*^2^ = 38%).

## Discussion

The specific aim of this systematic review was to compare failure rates of implants placed using a computer-generated guide and freehand placement. Guided placement resulted in accurate implant positioning in terms of parallelism between implants and less mesiodistal and buccolingual deviation.^6,42,44,45^ Although the accuracy in implant placement and survival rates using the two techniques have been reported extensively in the literature, there is limited information on implant failure rates with the two approaches.

Yogui et al. compared survival rates between computer-guided and freehand placement. They concluded that both techniques yielded a similar result.^48^ Also, Pozzi et al., in their review, suggested that survival rates of guided surgery were similar to conventional freehand protocols.^4^ According to a systematic review by Schneider et al., computer-guided implant placement had higher implant survival rates ranging from 91 to 100% after 12–60 months of follow-up.^49^

This systematic review found a significant difference in the failure rates between the two techniques. Eight studies met the inclusion criteria, and out of these, four were included in the meta-analysis. Meta-analysis indicated that the RR for guided implant placement was 0.29 (0.15,0.58) *p* < 0.001. The incidence of implant failure in freehand surgery was almost three times higher than the guided protocol. Despite the overall low heterogeneity (*I*^2^ = 38%) among included studies, some variations existed in the follow-up duration, secondary interventions, and implant surgery types. This is not an uncommon finding in systematic reviews.

The fully guided technique presents an advantage of accuracy in implant placement when compared to the freehand technique. An accurate implant placement ensures a predictable restorative outcome. One disadvantage of a fully guided approach is that it involves additional cost, and in cases of limited mouth opening, following a fully guided drill sequence can be challenging.^48^ In addition, few studies have reported that guided surgeries can easily cause operator oversight during osteotomy preparation resulting in inadequate irrigation during surgery.^48^ This could interfere with bone healing and compromise the outcome.^6,48^ However, the finding of this systematic review was contrary to the suggested outcome.

In addition, some studies compared intraoperative and postoperative complications and morbidity following implant placement.^46,47^ In contrast to other studies, the present study reported reduced postoperative morbidity in terms of swelling, pain, and bleeding with guided implant placement compared to the freehand approach.^4,43^ Proper case selection and surgical execution could contribute to these differences.

One limitation that could be noted from the included studies was the differences in the operators’ clinical experience and skill set. Another limitation was a low number of quality studies comparing guided to freehand implants. These observations call for the need for more standardized RCTs to establish the evidence, estimate the effect size, and standardize the potential effect modification in using guided and freehand implant placement techniques.

## Conclusion

Within the limitations of this systematic review, both guided and freehand implant placement have a high implant survival rate. However, based on the results, implant failure rates were almost three times higher in freehand placement.
